# The Association of Nevus-Associated Melanoma with Common or Dysplastic Melanocytic Nevus: A Systematic Review and Meta-Analysis

**DOI:** 10.3390/cancers15030856

**Published:** 2023-01-30

**Authors:** Clio Dessinioti, Aggeliki Befon, Alexander J. Stratigos

**Affiliations:** 1 1st Department of Dermatology-Venereology, Andreas Sygros Hospital, National and Kapodistrian University of Athens, 16121 Athens, Greece; 2 State Department of Dermatology-Venereology, Andreas Sygros Hospital, 16121 Athens, Greece

**Keywords:** cutaneous melanoma, nevus-associated, nevus, acquired, common, dysplastic

## Abstract

**Simple Summary:**

Acquired melanocytic nevi are classified as common or dysplastic. Cutaneous melanoma may develop in a pre-existing acquired nevus (nevus-associated melanoma, NAM). We conducted a systematic review and meta-analysis of 22 published articles to investigate whether NAM occurs more frequently within a dysplastic or common nevus. Although our meta-analysis showed a similar proportion of 51% dysplastic nevus (compared with common nevus) in NAM, when including those studies with larger patient numbers, there was a higher proportion of 65% NAM developing in a dysplastic nevus. A separate meta-analysis of invasive and *in situ* NAMs showed that the proportion of dysplastic nevus were 56% and 71%, respectively. The larger proportion of dysplastic nevus in *in situ* NAMs should be interpreted with caution due to the low numbers and possible misclassification bias of in situ NAM developing in dysplastic nevus that may be challenging to differentiate from a dysplastic nevus. Our meta-analysis had considerable uncertainty and high heterogeneity, highlighting the need for future well-designed studies with uniform histopathological definitions for dysplastic nevus remnants which report the type of nevus in NAM separately for invasive and *in situ* melanomas, thin tumors, and by histological subtype.

**Abstract:**

**Background**: Cutaneous melanoma has an adjacent nevus remnant upon histological examination in 30% of cases (nevus-associated melanoma, NAM), while it appears *de novo* for 70% of tumors. Regarding NAM arising in acquired melanocytic nevus, currently there is no evidence on whether NAM more frequently develops in association with a dysplastic or common melanocytic nevus. **Objectives**: To conduct a systematic review and meta-analysis to investigate the proportion of dysplastic or common melanocytic nevus in NAM associated with acquired nevus. **Methods**: A systematic literature search is conducted using PubMed, Scopus, and the Cochrane Library. The PRISMA checklist is used. Studies reporting patients diagnosed with NAM arising in an acquired common or dysplastic melanocytic nevus are included. A meta-analysis of proportions is performed using the random-effects model. The magnitude of heterogeneity is assessed with the I^2^ statistic. **Results**: A total of 22 studies with 2174 NAMs with an acquired nevus (dysplastic or common) are included. The proportion of dysplastic nevus in NAM varies considerably in the included studies, ranging from 0% to 100%. In the meta-analysis, the overall estimate of the proportion of having a dysplastic nevus in NAM is 51% (95% CI: 39–63%) with high heterogeneity at I^2^: 95.8% (*p* < 0.01). A sensitivity meta-analysis of 12 studies that included 30 or more acquired nevus-NAMs (2023 cases) shows that 65% of the NAMs developed in a dysplastic nevus (95% CI: 51–77%). In a meta-analysis of 4 studies reporting invasive-only acquired nevus-NAMs (764 cases), the proportion of dysplastic nevus is 56% (95% CI: 36–75%). Only 2 studies are found reporting *in situ* NAMs with an acquired nevus, and the pooled estimated proportion of dysplastic nevus is 71% (95% CI: 63–78%). **Conclusions**: The results of this meta-analysis suggest a higher proportion of dysplastic nevus in acquired nevus-NAM; however, there is considerable uncertainty and high heterogeneity, highlighting the need for future well-designed studies with uniform histopathological definitions for dysplastic nevus remnants which report the type of nevus in NAM separately for invasive melanomas, thin tumors, and by histological subtype.

## 1. Introduction

The relation between melanocytic nevi and melanoma is multifaceted. The number of acquired melanocytic nevi on the body, including common and dysplastic nevi, is an established marker of the risk of the development of cutaneous melanoma [1]. Moreover, the possibility of melanocytic nevi to act as precursors for the development of melanoma is more complex and intriguing. The presence of nevi in histological association with melanoma (nevus-associated melanoma, NAM) has fueled the debate on the malignant transformation of nevi to nevus-associated melanoma. Recent research has provided accumulating evidence on the distinct epidemiological, histological, dermoscopic, and genetic characteristics of NAM compared with melanoma developing *de novo* [2,3,4,5] even in thin melanomas [3], thus supporting a distinct route of stepwise development in NAM [6].

It is not known whether NAM is more likely to develop in a common or dysplastic acquired nevus. In their stepwise model of melanoma progression, Clark et al. proposed that if melanoma is to develop via a precursor lesion, the nevus with melanocytic dysplasia is that precursor [7]. A dysplastic nevus was described as a nevus with atypical melanocytic hyperplasia, melanocytic cytologic atypia, mesenchymal changes in the papillary dermis, and a lymphocytic infiltrate [8,9]. The WHO 2018 histological diagnosis of dysplastic nevi is based on the diagnostic criteria by the International Melanoma Pathology Study Group: (1) width > 4 mm; (2) architecture with irregular/dyscohesive nests of intraepidermal melanocytes and the increased density of non-nested junctional melanocytes; and (3) cytology with atypical melanocytes [10]. A modeling study by Tsao et al. that used population-based total melanocytic nevus counts estimated the malignant transformation rate of an individual nevus to be low and range from 0.00005% to 0.003% per year. However, it was noted that the estimation of the transformation rate of dysplastic nevi into melanoma was not possible due to the absence of the accurate documentation of dysplastic nevi age-specific density in a given individual [11].

We performed a systematic review and meta-analysis of the studies reporting the types of acquired nevus in association with NAM with the aim of answering the question of whether NAM is more likely to develop in association with a dysplastic or common acquired nevus. Such findings are useful to the monitoring follow-up and management of individuals with types of nevi more likely to evolve into melanoma as well as adding evidence regarding the previously proposed model of stepwise progression for melanoma development.

## 2. Methods

The Preferred Reporting Items for Systematic Reviews and Meta-Analyses (PRISMA) checklist was used to guide the project (Supplementary Table S1) [12]. The systematic review was prospectively registered with PROSPERO (registration number: CRD42022377596).

### 2.1. Literature Search and Study Selection

A systematic review of original articles investigating types of acquired melanocytic nevus in NAM was performed by searching in PubMed/MEDLINE, Scopus, and the Cochrane Library from 1 January 1980 through to 20 November 2022. The search strategy included the following keywords in various combinations: “melanoma”, “nevus”, “naevus”, ”nevi”, “naevi”, “acquired”, “dysplastic”, “nevus-associated”, “naevus-associated”. The full list of search terms is detailed in Supplementary Table S2. Inclusion criteria for eligible studies included: original studies reporting the histological association of cutaneous melanoma with the type of acquired nevus (common or dysplastic) in studies with at least 10 patients with NAM. Exclusion criteria were: NAM associated with congenital melanocytic nevus and studies not reporting the type of acquired melanocytic nevus remnants in NAM. We decided not to study congenital nevi and to instead focus on the research question of acquired nevus remnants (common or dysplastic) in NAM, considering the following: (1) The progression of melanoma from congenital nevus is not a debated issue, and the pathway of melanoma arising in congenital nevus is described in the current WHO classification of skin tumors [13]; (2) the risk of progression of congenital nevi to melanoma has been previously studied in individual studies and in systematic reviews [14,15]; (3) however, the development of melanoma within common or dysplastic nevus has not been previously studied in a systematic review and meta-analysis.

All languages were included in the search results. Non-English results were removed during the review process. Furthermore, the references of review articles on the topic were reviewed, and we carried out secondary referencing by manually reviewing reference lists of assessed articles. The authors of studies with missing results were contacted via email.

Two independent reviewers (C.D. and A.B.) selected the potential studies based on the inclusion and exclusion criteria for further assessment in full text and then screened the full texts, identified studies for inclusion, and recorded reasons for exclusion. Any disagreements were resolved by consensus or by consulting a third review author (A.J.S.). Duplicates were identified and removed. We completed a PRISMA flow diagram and a table with characteristics of excluded studies.

### 2.2. Data Extraction

Data were extracted and recorded using a standardized Excel sheet by two independent reviewers (C.D., A.B.). For each study, the following study characteristics were extracted: author, journal, year, study design, number of melanomas, number of nevus-associated melanomas (NAMs), number of NAMs associated with acquired melanocytic nevus, number of NAMs associated with common acquired nevus, number of NAMs associated with dysplastic nevus, age, sex, Breslow thickness of NAM, *in situ* or invasive NAM, and type of common nevus (junctional, dermal, or combined) in NAM. Any disagreements were resolved by consensus or by consulting a third review author (A.S.).

### 2.3. Quality Assessment

The quality of observational studies was assessed by two reviewers (C.D., A.S.) by assigning a risk of bias (high, moderate, or low) based on the limitations in the designs of included studies such as inconsistency and imprecision. Inconsistency was present in studies reporting NAM in non-relevant subgroups: invasive and *in situ* melanomas, various histological subtypes, or thin and thick melanomas. Imprecision was assessed based on the small number of participants of included studies [16]. The use of the Newcastle–Ottawa quality assessment scale (NOS) was not applicable as this concerns case–control or cohort studies [17].

### 2.4. Data Analysis

The primary outcome of interest was the proportion of NAM associated with a dysplastic nevus (compared with NAM associated with a common nevus). Meta-analysis of proportions was performed for the synthesis of single group data. The aim was to investigate the proportion of dysplastic or common melanocytic nevus in NAM associated with acquired nevus. Additional pre-planned analyses included a sensitivity meta-analysis excluding studies with a higher risk of bias, a meta-analysis including studies reporting the type of acquired melanocytic nevus, and a separate meta-analysis for invasive NAM. Post-hoc analyses included a meta-analysis stratified by geographical regions and stratified by year of publication (before and after 2000). Meta-analysis was performed with a random-effects model using the Metaprop command in Stata 12. Freeman and Tukey double arcsine transformation was applied [18]. Forest plots were constructed. The percentage of variation attributable to heterogeneity was assessed with the I^2^ statistic [19]. All statistical tests were two-sided and *p*-values less than 0.05 were considered statistically significant. The *p*-values reporting statistical significance are shown for the I^2^ statistic. The *p*-values for the estimated proportion in meta-analyses of proportions were not reported as they do not have any clinical significance; the null hypothesis test is the equality of the estimate with zero. The results were evaluated by effect size estimation in the point estimate and its confidence intervals. Analysis was conducted using STATA, version 12.0 (StataCorp LP, College Station, TX, USA).

## 3. Results

### 3.1. Included Studies

**Figure 1:**
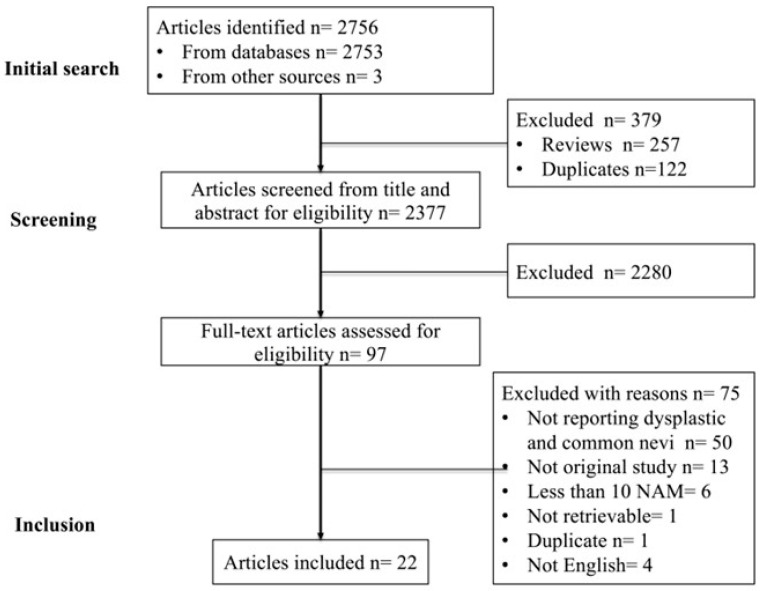
PRISMA flow diagram of studies identified, screened, and included in the meta-analysis.

A PRISMA flow diagram of study identification, screening, and inclusion is shown in Figure 1. The initial search identified a total of 2756 articles, including 2753 records from PubMed, Scopus, and Cochrane as well as a further 3 potentially eligible articles identified through secondary referencing. After excluding 257 reviews from automated processes in PubMed and 122 duplicates, 2377 records were screened for eligibility based on the titles and abstracts. A further 2280 non-eligible articles and non-English language articles from the titles and abstracts were excluded, and 97 potentially eligible articles were assessed from the full text. From these 97 articles, 75 articles were excluded (detailed in Supplementary Table S3 with reasons for exclusion). We contacted the authors of studies with missing results via email but did not receive any replies. As a result, a total of 22 studies were included [7,20,21,22,23,24,25,26,27,28,29,30,31,32,33,34,35,36,37,38,39,40]. Eight studies were assessed to be at low risk of bias, six studies were at moderate risk of bias, and eight studies were at high risk of bias (Supplementary Table S4).

### 3.2. Characteristics of NAM with an Acquired Nevus in Included Studies

The details of the 22 included studies are shown in Table 1. These studies included 3088 NAMs, of which 2174 were NAMs with an acquired nevus (common or dysplastic). All the studies were of a retrospective design. Regarding the inclusion of invasive or *in situ* melanomas, 8 studies included only invasive melanomas [21,22,24,27,29,30,32,33], 4 studies did not report whether the melanomas were invasive or *in situ* [7,23,25,26], and 10 studies included invasive and *in situ* melanomas [20,28,31,34,35,36,37,38,39,40]. Of the 10 studies that included invasive as well as *in situ* melanomas, 4 studies included both invasive and *in situ* melanomas without reporting the case numbers [34,35,36,39], 4 studies reported the number of invasive and *in situ* melanomas without specifying the nevus remnant type [20,28,37,38], and 2 studies reported the number of invasive and in situ melanomas and specified the nevus remnant type in in situ NAMs [31,40] (Table 1).

Of the 22 studies, 12 studies included 30 or more NAMs with an acquired nevus. The mean or median Breslow thickness of the NAMs were reported in only 9 studies (mean Breslow range: 0.51–1.38 mm and median Breslow range: 0.35–2.3 mm, Table 1).

The histologic subtypes of NAM were reported in 15 studies, of which one study had all preselected SSM and one study had all preselected NM. In the remaining 13 studies, the most common subtypes were SSM and NM, and the percentage of SSM ranged from 50% to 100%, while the percentage of NM ranged from 0 to 13% (Table 1).

The type of common nevus in NAM was reported in eight studies, of which three studies used the histological criteria described by Ackerman [41,42], classifying acquired nevi as Clark’s nevi, Miescher’s nevi, and Unna’s nevi. Miescher’s nevi present as dome- or wedge-shaped lesions with mostly endophytic distributed melanocytes, and Unna’s nevi are exophytic, papillomatous lesions confined to a thickened papillary dermis. Unna and Miescher are classified as intradermal [23] (Table 1).

The histopathological criteria for dysplastic nevus remnants in NAM were variably defined in the included studies and are shown in Table 2.

### 3.3. Meta-Analysis of the Proportion of Dysplastic or Common Acquired Nevus in NAM Studies (n = 22)

**Figure 2:**
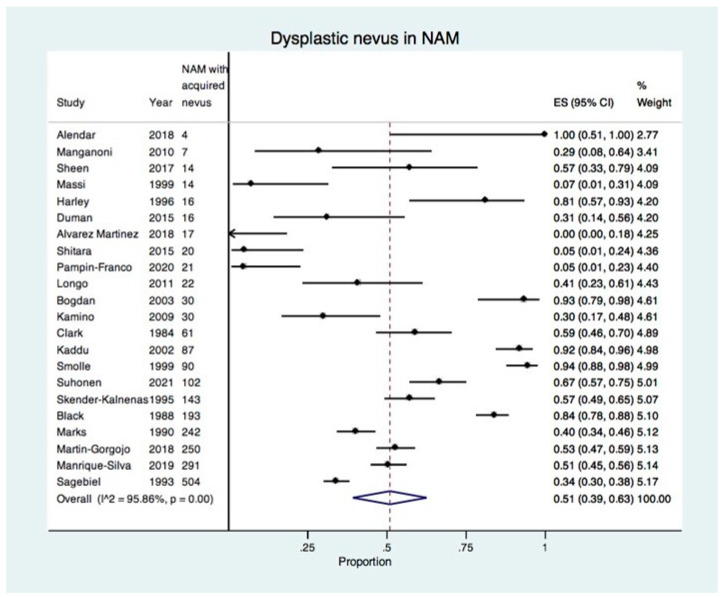
Forest plot showing the proportion of dysplastic nevus in all included studies reporting NAM developing in an acquired nevus (22 studies, 2174 NAMs with an acquired nevus) [7,20,21,22,23,24,25,26,27,28,29,30,31,32,33,34,35,36,37,38,39,40].

There were 22 studies reporting NAM associated with a dysplastic or common acquired nevus [7,20,21,22,23,24,25,26,27,28,29,30,31,32,33,34,35,36,37,38,39,40] and the overall estimate of the proportion of a dysplastic nevus in NAM was 51% (95% CI: 39–63%) as shown in the forest plot in Figure 2. The estimate of the proportion of a dysplastic nevus in NAM was stratified according to the risk of bias (low, moderate, high) (Supplementary Figure S1). The proportion of a dysplastic nevus in NAM was higher in studies with a low risk of bias (60%, 95% CI: 45–73%) compared with studies with a high risk of bias (38%, 95% CI: 13–66%).

Overall, there was high heterogeneity with I^2^ of 95.86%, which was statistically significant (*p* < 0.01). This was due to the wide variation in the proportion of dysplastic nevus in NAM in the included studies, ranging from 0% to 100% (Table 1). In order to increase precision and to explore heterogeneity, a sensitivity meta-analysis was performed for studies reporting 30 or more NAMs with an acquired nevus.

### 3.4. Sensitivity Meta-Analysis of Studies Including 30 or More NAMs with an Acquired Nevus (n = 12)

**Figure 3:**
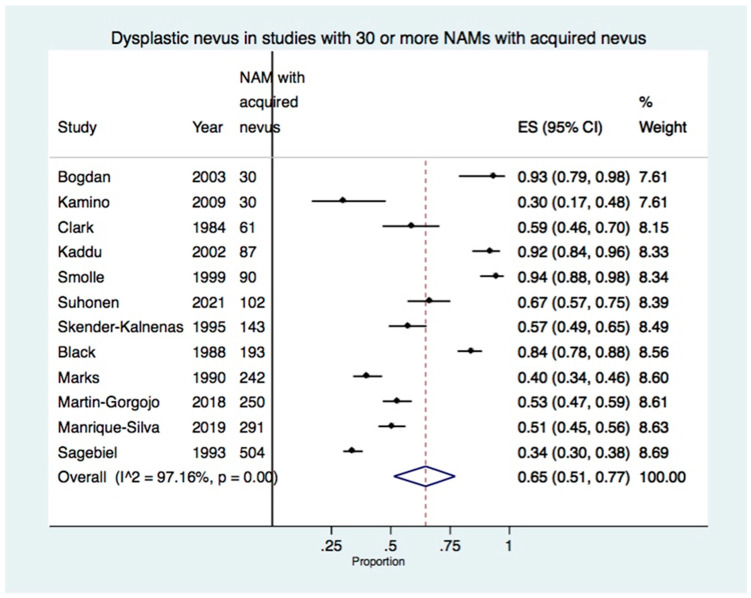
Forest plot showing the proportion of dysplastic nevus in studies with 30 or more NAMs developing in an acquired nevus (12 studies, 2023 NAMs with an acquired nevus) [7,22,23,26,27,30,31,32,35,38,39,40].

There were 12 studies reporting 30 or more NAMs with an acquired nevus, including a total of 2023 cases [7,22,23,26,27,30,31,32,35,38,39,40] (Table 1). The meta-analysis of these studies showed the presence of dysplastic nevus in 65% of acquired nevus-NAM (95% CI: 51–77%) (Figure 3). There was high heterogeneity with I^2^: 97.16% (*p* < 0.01), which was due to the variability of the proportion of dysplastic nevus in the included studies, ranging from 30% to 94.4% (Table 1).

When the study of Suhonen et al. which had a large number of missing values in the presence or absence of nevus in melanoma was additionally excluded from the meta-analysis, the results remained similar, with dysplastic nevus present in 65% of NAM (95% CI: 50–78%).

### 3.5. Sensitivity Meta-Analysis of Studies Including Invasive-Only NAM

There were eight studies including invasive-only acquired nevus-NAMs [21,22,24,27,29,30,32,33] (Table 1). In the meta-analysis, the estimated proportion of dysplastic nevus in invasive acquired nevus-NAM was 36% (95% CI: 19–55%) (Supplementary Figure S2). However, there were studies with a very small number of acquired nevus-NAMs, ranging from 7 NAMs in a study by Manganoni et al. to 17 NAMs in a study by Alvarez Martinez et al. Moreover, the study by Alvarez Martinez et al. reported 0 cases of dysplastic nevus NAM, while a study by Massi et al. reported 7% of 14 NAMs developed in a dysplastic nevus.

**Figure 4:**
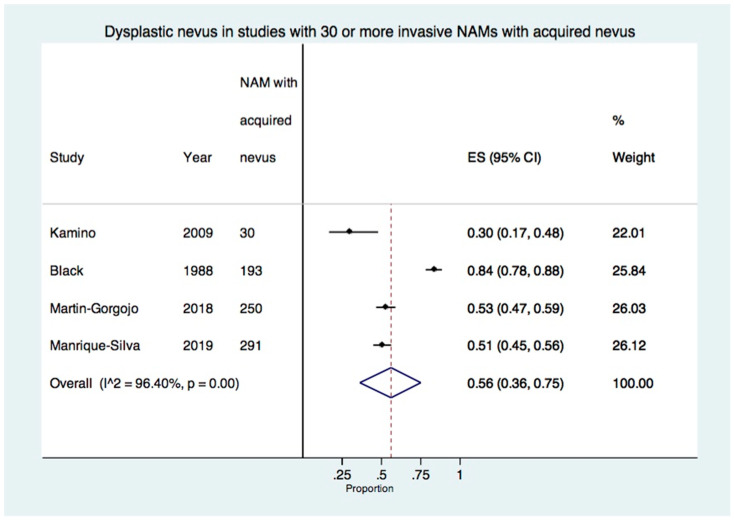
Forest plot of the proportion of dysplastic nevus in studies with 30 or more invasive-only acquired nevus NAMs (4 studies, 764 invasive-only acquired nevus NAMs) [22,27,30,32].

When excluding studies with less than 30 acquired nevus-NAMs, 4 studies with a total of 764 invasive acquired nevus-NAMs were included in the meta-analysis [22,27,30,32]. The estimated proportion of dysplastic nevus in invasive NAM was 56% (95% CI: 36–75%). There was a high degree of heterogeneity at 96.4% (*p* < 0.01) (Figure 4).

### 3.6. Sensitivity Meta-Analysis of Studies Including In Situ NAM

Regarding *in situ* acquired nevus-NAMs, there were only 2 studies reporting acquired nevus remnant type [31,40]; Marks et al. reported 50/85 *in situ* dysplastic nevus NAM (59%) [31], and Suhonen et al. reported 46/53 *in situ* dysplastic nevus NAM (87%) [40]. In a meta-analysis of these 2 studies, there was a pooled proportion of 71% (95% CI: 63–78%) dysplastic nevus in *in situ* NAMs.

### 3.7. Meta-Analysis Stratified by Region

There were six studies including patients from Southern Europe [28,29,30,32,33,34]; six studies from Northern/Western Europe [20,21,23,26,39,40]; five studies from North America [7,22,25,27,35]; two studies from Australia [31,38]; one study from Eastern Asia [36]; one study from Western Asia [24]; and one study from Brazil and Austria [37] that could not be classified into one region.

In a meta-analysis stratified by region, the proportion of dysplastic nevus NAM was 46% (95% CI: 41–51%) for Australia, 58% (95% CI: 30–84%) for North America, 76% (95% CI: 49–96%) for Northern/Western Europe, and 32% (95% CI: 19–47%) for Southern Europe (Supplementary Figure S3).

**Figure 5:**
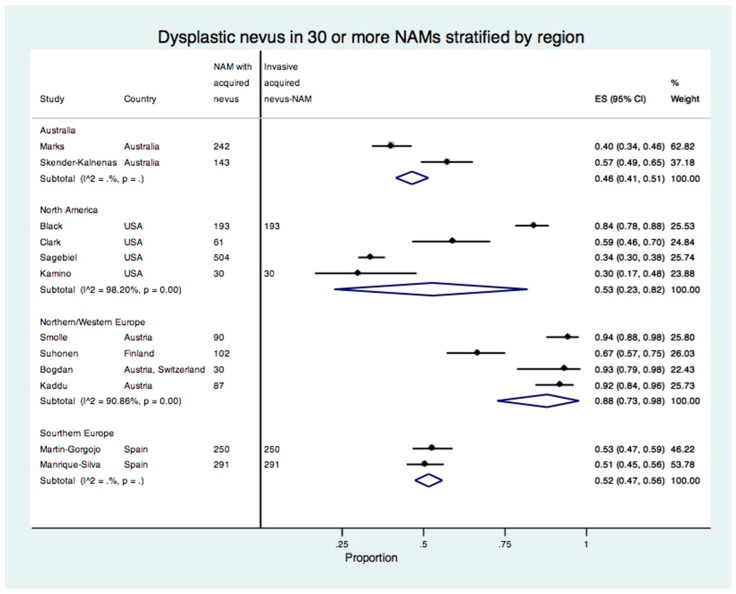
Forest plot of the proportion of dysplastic nevi in studies with 30 or more acquired nevus NAMs stratified by region.

In a meta-analysis of studies including 30 or more acquired nevus-NAMs stratified by region, the proportion of dysplastic nevus NAM remained the same for Australia, 53% (95% CI: 23–82%) for North America, 88% (95% CI: 73–98%) for Northern/Western Europe, and 52% (95% CI: 47–56%) for Southern Europe (Figure 5). The studies from Northern/Western Europe all reported a higher proportion of dysplastic nevi in NAM. By contrast, the studies from the USA showed discrepant results with the proportion of dysplastic nevus in NAM ranging from 30% to 84%. Only four studies reported the number of invasive acquired nevus NAMs (Figure 5).

## 4. Discussion

It is unclear whether individual dysplastic nevi progress to melanoma at higher rates than banal common nevi. Prospective studies are hindered by the need for long follow-ups and by the overall low rate of nevi transformation. Published studies have indirectly addressed this question by comparing the frequency of NAMs developing in remnants of dysplastic nevi versus the remnants of common nevi with variable and conflicting results. In order to investigate whether NAM occurs more frequently with a dysplastic or a common nevus, we performed a systematic review and meta-analysis of studies reporting NAM in acquired nevi. Although our meta-analysis of 22 studies (*n* = 2174) showed a similar proportion of 51% of a dysplastic nevus (compared with common nevus) in NAM, when including the 12 studies with 30 or more NAMs with an acquired nevus (*n* = 2023), there was a higher proportion of NAM developing in a dysplastic nevus at 65%. The separate meta-analysis of invasive and *in situ* NAMs showed that the proportions of dysplastic nevus were 56% and 71%, respectively.

The numbers of *in situ* and invasive NAMs included in the individual studies may have influenced the proportion of the detected dysplastic nevus remnant component. In our meta-analysis, in 4 studies including 30 or more invasive-only acquired nevus-NAMs (764 cases), the proportion of dysplastic nevus was 56% (95% CI: 36–75%). However, in 2 available studies that reported *in situ* NAMs, (138 cases), the proportion of dysplastic nevus was 71% (95% CI: 63–78%). These results should be interpreted with caution due to the small numbers and possible misclassification bias of *in situ* NAMs. The larger proportion of dysplastic nevus in *in situ* melanomas may indicate some misclassification arising from upgrading the histologic diagnosis of dysplastic nevus to melanomas *in situ* [50]. This “diagnostic drift” may arise from the pressure of medical liability and the variability in and challenges to the histopathological diagnosis of dysplastic nevi versus melanoma *in situ* [51,52]. The addition of dermoscopy to contribute to the diagnosis of melanoma *in situ* may aid in decreasing the misclassification of these lesions [53], while reflectance confocal microscopy may further aid correct diagnosis [34]. In addition, the dermoscopic characteristics of NAM compared with *de novo* melanoma have been described. Reiter et al. showed that NAMs were 2.5 times more likely to show a negative pigment network compared with *de novo* melanomas, even though the nevus component of NAM could not be identified dermoscopically [4]. Regarding the nevus component of NAM, Zalaudek et al. reported dermoscopic characteristics of congenital versus noncongenital NAMs, and in their series, the nevus component was mostly evident and characterized by regular dots/clods and structureless brown areas [54].

In our meta-analysis, there was high heterogeneity due to the variability of the proportion of dysplastic nevus in individual studies. This variability could be attributed mostly to differences in the included melanomas regarding the invasive or *in situ* type as well as the variable criteria used for the histological definition of dysplastic nevus remnants. It could also possibly be attributed to differences in the Breslow thickness and histological subtypes. It has been shown that the proportion of NAM is higher in thinner melanomas while thicker tumors may obliterate the nevus remnants [3,30], and NAM is more frequent in SSM compared with other subtypes [3,6,30,32]; however, it is not known whether the development of NAM with a common or dysplastic nevus could be different depending on the thickness of the melanoma or the histological subtype. In our systematic review, only the study by Martin-Gorgojo et al. analyzed dysplastic nevus NAM versus common nevus NAM and reported no significant differences in Breslow thickness or subtype [32]. Future studies should report the nevus remnant type in NAM separately for thin melanomas and if possible by histological subtype so that more clear evidence may accumulate.

Our meta-analysis stratified by region showed a higher proportion of dysplastic nevi in NAM in studies from Northern/Western Europe and discrepant estimates in individual studies from North America. These results may be explained by the differences in the number of invasive or *in situ* melanomas in individual studies. Regarding environmental and genetic factors, NAMs are more likely than *de novo* melanomas to be located on non-chronically sun-damaged skin, such as on the trunk and extremities [38,55], which in turn is more frequently associated with *BRAF* mutations [55,56]. In their landmark genetic study of 37 primary melanomas and their adjacent nonmalignant melanocytic neoplasms, Bastian and colleagues investigated the genetic evolution of melanomas from nevi, the sequence of mutations in melanocytic neoplasms, and a stepwise model of mutations [5]. The burden of point mutations escalated with each histologic stage, from benign nevi to intermediate lesions, melanoma *in situ*, and to invasive melanoma [5,57]. The study by Martin-Gorgojo et al. of 250 invasive NAMs reported no significant differences between common nevus NAM and dysplastic nevus NAM for age, sex, phototype, hair color, location, total body nevus counts, *BRAF*, *NRAS* mutations, or *MC1R* RHC gene variants [32]. Compared with dysplastic nevus NAM, common nevus NAM was more likely to have mitoses present [32]. Future studies may further investigate whether genetic and environmental factors differentially modulate the risk of NAMs developing in a dysplastic or common nevus.

The limitations of our meta-analysis pertain to the limitations of the included studies. Inconsistency underscores the need for studies in relevant patient/melanoma subgroups and for investigating separately *in situ* and invasive melanomas. Imprecision underscores the need for more studies that report the type of associated nevus in NAM. Our systematic review and meta-analysis examined the frequency of dysplastic nevus versus common nevus in NAM to help improve our understanding of the existing evidence on the potential of nevus transformation to melanoma. However, most existing studies do not distinguish between severe dysplasia and mild or moderate dysplasia, and it is not known if it would be correct to assume a uniform rate of the transformation of dysplastic nevi to melanoma [58]. An additional limitation was that eight studies had a low risk of bias while six had a moderate risk and eight studies had a high risk of bias. Moreover, the very small sample size of some of the included studies may have influenced the accuracy of the estimated proportion. The estimation is likely to be more precise in the meta-analysis including studies with 30 or more NAMs, which supports a pooled proportion of 65% dysplastic nevus in NAM, taking into consideration the fact that the reporting of *in situ* and invasive NAMs together may have contributed to these results.

The clinical implications of the possibility of nevi progressing to NAM are not clear. Congenital melanocytic nevi are recognized precursors of melanoma arising in congenital nevus, that is a melanoma subtype in the current WHO classification associated with a distinct pathway of development [10,14,15]. By contrast, acquired nevi are frequent, growth-arrested, clonal neoplasms of melanocytes [59]. Even though NAM represents 30% of melanomas [60,61], an acquired nevus rarely progresses to melanoma. The decision regarding when to follow up versus excise clinically atypical nevi incorporates findings with dermoscopy (dermatoscopy, epiluminescence microscopy, or skin surface microscopy) and, in high-risk patients, total-body photography [9]. Investigating the genetics of dermoscopic nevus patterns may shed further light on dysplastic nevi more likely to evolve into melanoma [62].

## 5. Conclusions

In conclusion, this systematic review synthesized what is known on the frequency of a dysplastic or common nevus associated with NAM, addressing the question of the development of NAM within an acquired nevus. The results of our meta-analysis suggest that a higher proportion of acquired nevus-NAMs develop within a dysplastic nevus (65%); however, there was considerable uncertainty (95% CI: 51–77%) and high heterogeneity, highlighting the need for future well-designed studies with uniform histopathological definitions for dysplastic nevus remnants which report the type of nevus in NAM separately for invasive and *in situ* melanomas, thin tumors, and by histological subtype.

## Figures and Tables

**Table 1:** Included studies reporting the type of acquired melanocytic nevus in histological association with cutaneous melanoma (*n* = 22).

Study, Year	NAM, n (Invasive)	Mean Breslow NAM, mm	Subtype NAM, n	Dysplastic Nevus in NAM, n	Common Nevus in NAM, n	Acquired Nevus in NAM, n	Dysplastic Proportion in Acquired (Dysplastic + Common)	Common Nevus Type in NAM			Comment
								J, n	D, n	C, n	
Alendar, 2018 [20]	31 (26)	Median 0.85	NR	4	0	4	100%	0	0	0	Consecutive patients
Alvarez Martinez, 2018 [21]	20 (all)	Median: 0.35	SSM: 20	0	17	17	0%	2	15	0	Preselected invasive melanomas with 2 follow-up images
Black, 1988 [22]	211 (all)	NR	SSM: all	162	31	193	83.9%	0	23	8	Invasive SSMs
Bogdan, 2003 [23]	30 (NR)	NR	SSM: 26 NM: 4	Clark’s: 28 *	2 *	30	93.3%		M: 1 U: 1		Preselected NAM
Clark, 1984 [7]	74 (NR)	NR	SSM: 59 NM: 6 LMM: 5 ALM: 0 NOS: 4	36	25	61	59%	NR	NR	NR	Consecutive patients
Duman, 2015 [24]	28 (28)	Median: 1.8	NR	5	11	16	31.3%	NR	NR	NR	
Harley, 1996 [25]	29 (NR)	Mean: 1.38	NR	13 *	3 *	16	81.3%		M: 2 U: 1		Consecutive patients
Kaddu, 2002 [26]	148 (NR)	Mean: 1.3	NR	80	7	87	92%	NR	NR	NR	Consecutive patients
Kamino, 2009 [27]	30 (all)	NR	SSM: 27 NM: 3	9	21	30	30%	NR	NR	NR	Preselected NAM of SSM or NM subtype
Longo, 2011 [28]	33 (23)	Median: 0.43	NR	9	13	22	40.9%	0	5	8	Consecutive patients with confocal images
Manganoni, 2010 [29]	10 (all)	Median 2.3	Preselected NM	2	5	7	28.6%	NR	NR	NR	Consecutive patients with NM
Manrique-Silva, 2019 [30]	291 (all)	NR	SSM: 252 NM: 39	147	144	291	50.5%	NR	NR	NR	Patients with invasive SSM or NM and NAM with common or dysplastic nevi
Marks, 1990 [31]	257 (168)	NR	SSM: 254 NM: 3	97	145	242	40.1%	NR	NR	NR	Patients with SSM or NM
Martin-Gorgojo, 2018 [32]	250 (all)	NR	SSM: 211 NM: 29 LMM: 4 ALM: 3 NOS: 2	132	118	250	52.8%	NR	NR	NR	Invasive melanoma, NAM with common or dysplastic nevi
Massi, 1999 [33]	27 (all)	NR	SSM: 25 NM: 2	1	13	14	7.1%	NR	NR	NR	Consecutive patients with invasive melanoma
Pampin-Franco, 2020 [34]	22 (both #)	Mean: 0.51	SSM: 20 LMM: 1 NOS: 1	1	20	21	4.8%	NR	NR	NR	Selected NAM with dermoscopy and confocal images
Sagebiel, 1993 [35]	1126 (both #)	NR	SSM: 714 NM: 94 Missing: 318	171	333	504	33.9%	NR	NR	NR	Consecutive patients with SSM or NM
Sheen, 2017 [36]	18 (both #)	NR	SSM: 9 NM: 2 ALM: 7	8	6	14	57.1%	NR	NR	NR	Consecutive patients in Asia
Shitara, 2015 [37]	61 (40)	NR	SSM: 39 NM: 1 ALM: 1 NOS: 18 Missing: 2	1	19	20	5%	6	3	10	Preselected NAM
Skender-Kalnenas, 1995 [38]	147 (93)	NR	SSM: 37 LMM: 2 Others: 8 Missing:100	82	61	143	57.3%	NR	NR	NR	Thin melanomas
Smolle, 1999 [39]	143 (both #)	Mean: 0.95	NR	85 *	5 *	90	94.4%		M: 3 U: 2		Consecutive melanoma biopsies
Suhonen, 2021 [40]	102 (49)	NR	NR	68	34	102	66.6%	NR	NR	NR	In 337 melanomas, information for nevus presence in 146 (43.3%)

*n*: number, NAM: nevus-associated melanoma, SSM: superficial spreading melanoma, NM: nodular melanoma, LMM: lentigo maligna melanoma, ALM: acral lentiginous melanoma, NOS: not otherwise specified, NR: not reported, J: junctional, D: dermal, C: compound, M: Miescher, U: Unna. # Both invasive and in situ NAMs were included, but the percentage of invasive tumors was not reported. * Classified according to the histological criteria described by Ackerman.

**Table 2:** Histopathological criteria for dysplastic nevus remnants in nevus-associated melanoma as defined in included individual studies.

Study	Criteria *
Alendar [20]	The nevus classification according to Ackerman [42].
Alvarez Martinez [21]	Agreement of at least two expert dermatopathologists. Nevus remnants assessed using previously defined criteria such as an abrupt transition between benign melanocytic cells and the adjacent malignant cells, the presence of maturation, and the absence of cytologic atypia within the nevic component [43].
Black [22]	Required several cross sections through the tumor and at minimum a 2 mm width of cutaneous tissue uninvolved by melanoma at two or more margins. Diagnosis of melanocytic dysplasia based on well-described criteria [7,44,45,46], including hyperplasia of melanocytes, cytologic atypia of melanocytes, and host response within the papillary dermis.
Bogdan [23]	Acquired nevi associated with melanoma fulfilled the histological criteria described by Ackerman [41,42].
Clark [7]	Melanocytic nevi with dysplasia had five distinctive histologic features: (1) persistent lentiginous melanocytic hyperplasia, (2) atypical melanocytic hyperplasia (melanocytic nuclear atypia), (3) lamellar fibroplasia, (4) concentric eosinophilic fibroplasia, and (5) sparse, patchy lymphocytic infiltrates.
Duman [24]	Sections reassessed by a dermatologist and a pathologist. No criteria reported.
Harley [25]	Independent review by two dermatopathologists. Disagreements were resolved by a third dermatopathologist. Dysplastic nevi in histologic contiguity were diagnosed by the following criteria: disordered intraepidermal melanocytic proliferation in lentiginous and/or junctional nested patterns, tendency of confluence of cells or theques along the basal layer, variable nuclear atypia of melanocytes, epidermal hyperplasia in the pattern of a lentigo, papillary dermal fibroplasia in concentric or lamellar patterns, and patchy perivascular or bandlike lymphocytic infiltrates. There had to be a recognizable difference in degree of nuclear atypia between the adjacent dysplastic component and the main part of the melanoma.
Kaddu [26]	At least eight sections were reviewed in each case. Nevus remnants were recognized as distinct from melanoma and further categorized into acquired and congenital types based on well-accepted morphological characteristics (see Table 1 of Kaddu et al. for details).
Kamino [27]	An associated nevus was defined as a distinct second population of small, uniform melanocytes without cytologic atypia adjacent to and/or beneath the malignant melanoma.
Longo [28]	Review by two board-certified pathologists blinded to the final diagnosis. The location of the nevus in relation to the melanoma and its contiguity or clear separation was taken into account.
Manganoni [29]	Not reported.
Manrique-Silva [30]	Not reported.
Marks [31]	Virtually all lesions were processed in their entirety. Nevi in NAM were classified as common acquired, dysplastic, or congenital [47,48]. Borderline tumors were examined by two or more pathologists before being classified.
Martin-Gorgojo [32]	Dysplastic or Clark nevi were defined based on the pathology subgroup of the European Organization for Research and Treatment of Cancer (EORTC) Malignant Melanoma Cooperative Group diagnostic criteria [49].
Massi [33]	Independent review by three pathologists blinded to the original diagnosis. Dysplastic nevi were diagnosed in the presence of histopathological criteria by Clark et al. [7,47]: (1) persistent lentiginous melanocytic hyperplasia, (2) atypical melanocytic hyperplasia (melanocytic nuclear atypia), (3) lamellar fibroplasia, (4) concentric eosinophilic fibroplasia, and (5) sparse patchy lymphocytic infiltrate.
Pampin-Franco [34]	Not reported.
Sagebiel [35]	Dysplastic nevi were defined by criteria including: lateral extension of an intraepidermal melanocytic proliferation beyond a dermal nevus; disordered architecture of the epidermis; a cellular-mesenchymal host response, including angiogenesis, fibroplasia, and cross-bridging of adjacent rete ridges; and the presence of atypical individual melanocytes in the epidermis. Because of the characteristic pattern of the epidermal ridge pattern and junctional nest cross-bridging in dysplastic nevi, the junctional changes were considered an integral part of the diagnosis.
Sheen [36]	Reviewed by at least 2 pathology faculty members at diagnosis.
Shitara [37]	New slides from cases of NAMs were independently evaluated by experienced dermatopathologists and a dermatologist. The nevus cytology criteria used for differentiation from melanoma cells were described in Veronese et al., J Bras Patol Med Lab 2006; 42:375–93, Veronese et al., J Bras Patol Med Lab 2007; 43:363–8, Baruhill R. Textbook of Dermatopathology. United States of America: McGraw-Hill, 1998.
Skender-Kalnenas [38]	Dysplastic nevi were defined according to the criteria of Elder et al. [47]. Junctional dysplastic nevi were distinguished from melanomas in situ by the presence of random, non-confluent, cytologic atypia and by the absence of pagetoid invasion of the overlying epidermis.
Smolle [39]	Acquired nevi were classified as Unna’s, Miescher’s, or Clark’s according to Ackerman [41,42]. Clark’s was defined as a slightly domed lesion with nests of melanocytes at the dermoepidermal junction and within a thickened papillary dermis, with the junctional component extending beyond the intradermal component.
Suhonen [40]	Not reported.

* The references in the definitions of histopathological criteria are presented as cited in the individual studies.

## Supplementary Materials

The following supporting information is available at https://www.mdpi.com/article/10.3390/cancers15030856/s1: Supplementary Table S1. PRISMA 2020 Checklist; Supplementary Table S2. Search terms in PubMed; Supplementary Table S3. Reasons for exclusion of fully assessed articles; Supplementary Table S4. Assessment of risk of bias; Supplementary Figure S1. Forest plot of the proportion of dysplastic nevus in NAM stratified by the risk of bias of individual studies; Supplementary Figure S2. Forest plot of the proportion of dysplastic nevus in studies with invasive-only acquired nevus-NAM; Supplementary Figure S3. Forest plot of the proportion of dysplastic nevus in acquired nevus-NAM stratified by region.
