# Supplementary material for: The Association of Nevus-Associated Melanoma with Common or Dysplastic Melanocytic Nevus: A Systematic Review and Meta-Analysis
Source: Cancers (Basel). 2023 Jan 30;15(3):856. doi: 10.3390/cancers15030856 (PMC9913707; doi:10.3390/cancers15030856)
Supplement: Supplementary file 1 [file cancers-15-00856-s001.zip › cancers-2176252-supplementary.pdf]

Supplementary Tables & Figures

Supplementary Table S1. PRISMA 2020 Checklist

Section and Topic	Item #	Checklist item	Page
**TITLE**			
Title	1	Identify the report as a systematic review.	1
**ABSTRACT**			
Abstract	2	See the PRISMA 2020 for Abstracts checklist.	1, 2
**INTRODUCTION**			
Rationale	3	Describe the rationale for the review in the context of existing knowledge.	2
Objectives	4	Provide an explicit statement of the objective(s) or question(s) the review addresses.	2
**METHODS**			
Eligibility criteria	5	Specify the inclusion and exclusion criteria for the review and how studies were grouped for the syntheses.	3
Information sources	6	Specify all databases, registers, websites, organisations, reference lists and other sources searched or consulted to identify studies. Specify the date when each source was last searched or consulted.	2
Search strategy	7	Present the full search strategies for all databases, registers and websites, including any filters and limits used.	Table S2
Selection process	8	Specify the methods used to decide whether a study met the inclusion criteria of the review, including how many reviewers screened each record and each report retrieved, whether they worked independently, and if applicable, details of automation tools used in the process.	3
Data collection process	9	Specify the methods used to collect data from reports, including how many reviewers collected data from each report, whether they worked independently, any processes for obtaining or confirming data from study investigators, and if applicable, details of automation tools used in the process.	3
Data items	10a	List and define all outcomes for which data were sought. Specify whether all results that were compatible with each outcome domain in each study were sought (e.g. for all measures, time points, analyses), and if not, the methods used to decide which results to collect.	3, 4
	10b	List and define all other variables for which data were sought (e.g. participant and intervention characteristics, funding sources). Describe any assumptions made about any missing or unclear information.	3, 4
Study risk of bias assessment	11	Specify the methods used to assess risk of bias in the included studies, including details of the tool(s) used, how many reviewers assessed each study and whether they worked independently, and if applicable, details of automation tools used in the process.	3, Table S4

Section and Topic	Item #	Checklist item	Page
Effect measures	12	Specify for each outcome the effect measure(s) (e.g. risk ratio, mean difference) used in the synthesis or presentation of results.	3, 4
Synthesis methods	13a	Describe the processes used to decide which studies were eligible for each synthesis (e.g. tabulating the study intervention characteristics and comparing against the planned groups for each synthesis (item #5)).	6-7
	13b	Describe any methods required to prepare the data for presentation or synthesis, such as handling of missing summary statistics, or data conversions.	n/a
	13c	Describe any methods used to tabulate or visually display results of individual studies and syntheses.	3
	13d	Describe any methods used to synthesize results and provide a rationale for the choice(s). If meta-analysis was performed, describe the model(s), method(s) to identify the presence and extent of statistical heterogeneity, and software package(s) used.	3
	13e	Describe any methods used to explore possible causes of heterogeneity among study results (e.g. subgroup analysis, meta-regression).	3
	13f	Describe any sensitivity analyses conducted to assess robustness of the synthesized results.	3
Reporting bias assessment	14	Describe any methods used to assess risk of bias due to missing results in a synthesis (arising from reporting biases).	3
Certainty assessment	15	Describe any methods used to assess certainty (or confidence) in the body of evidence for an outcome.	3
**RESULTS**			
Study selection	16a	Describe the results of the search and selection process, from the number of records identified in the search to the number of studies included in the review, ideally using a flow diagram.	4, Fig 1
	16b	Cite studies that might appear to meet the inclusion criteria, but which were excluded, and explain why they were excluded.	4, Table S3
Study characteristics	17	Cite each included study and present its characteristics.	4,5, Table 1
Risk of bias in studies	18	Present assessments of risk of bias for each included study.	Table S4
Results of individual studies	19	For all outcomes, present, for each study: (a) summary statistics for each group (where appropriate) and (b) an effect estimate and its precision (e.g. confidence/credible interval), ideally using structured tables or plots.	Table 1, Fig 2
Results of syntheses	20a	For each synthesis, briefly summarise the characteristics and risk of bias among contributing studies.	5-13, Table S4
	20b	Present results of all statistical syntheses conducted. If meta-analysis was done, present for each the summary estimate and its precision (e.g. confidence/credible interval) and measures of statistical heterogeneity. If comparing groups, describe the direction of the effect.	9-13, Fig 2, Fig 3, Fig 4, Fig 5
	20c	Present results of all investigations of possible causes of heterogeneity among study results.	10-13, Fig 3, Fig 4, Fig 5, Fig S1, Fig S2,

Section and Topic	Item #	Checklist item	Page
			Fig S3
	20d	Present results of all sensitivity analyses conducted to assess the robustness of the synthesized results.	10-13
Reporting biases	21	Present assessments of risk of bias due to missing results (arising from reporting biases) for each synthesis assessed.	n/a
Certainty of evidence	22	Present assessments of certainty (or confidence) in the body of evidence for each outcome assessed.	9-13, Fig 2, Fig 3, Fig 4, Fig 5 Fig S1, Fig S2, Fig S3
**DISCUSSION**			
Discussion	23a	Provide a general interpretation of the results in the context of other evidence.	13
	23b	Discuss any limitations of the evidence included in the review.	14, 15
	23c	Discuss any limitations of the review processes used.	14, 15
	23d	Discuss implications of the results for practice, policy, and future research.	15
**OTHER INFORMATION**			
Registration and protocol	24a	Provide registration information for the review, including register name and registration number, or state that the review was not registered.	2
	24b	Indicate where the review protocol can be accessed, or state that a protocol was not prepared.	2
	24c	Describe and explain any amendments to information provided at registration or in the protocol.	3, 4
Support	25	Describe sources of financial or non-financial support for the review, and the role of the funders or sponsors in the review.	16
Competing interests	26	Declare any competing interests of review authors.	16
Availability of data, code and other materials	27	Report which of the following are publicly available and where they can be found: template data collection forms; data extracted from included studies; data used for all analyses; analytic code; any other materials used in the review.	16

From: Page MJ, McKenzie JE, Bossuyt PM, Boutron I, Hoffmann TC, Mulrow CD, et al. The PRISMA 2020 statement: an updated guideline for reporting systematic reviews. BMJ 2021;372:n71. doi: 10.1136/bmj.n71

For more information, visit: http://www.prisma-statement.org/

Supplementary Table S2. Search terms in PubMed

((((((((((((((((((("1980/01/01"[Date - Publication] : "3000"[Date - Publication])) AND (((melanoma[Title/Abstract]) AND ((mole[Title/Abstract] OR nevus[Title/Abstract] OR naevus[Title/Abstract] OR nevi[Title/Abstract] OR naevi[Title/Abstract] OR nevomelanocytic[Title/Abstract]))) AND ((remnant[Title/Abstract] OR nevus-associated[Title/Abstract] OR naevus-associated[Title/Abstract])))) OR (((melanoma[Title/Abstract]) AND (nevomelanocytic[Title/Abstract])) AND ((remnant[Title/Abstract] OR arise[Title/Abstract] OR arose[Title/Abstract] OR association[Title/Abstract] OR associated[Title/Abstract])))) OR (((melanoma[Title/Abstract]) AND (mole[Title/Abstract])) AND (associated[Title/Abstract]))) OR ((melanoma[Title/Abstract]) AND (common nevi[Title/Abstract]))) OR ((melanoma[Title/Abstract]) AND (dysplastic naevi[Title/Abstract]))) OR ((melanoma[Title/Abstract]) AND (dysplastic nevi[Title/Abstract]))) OR ((melanoma[Title/Abstract]) AND (remnants[Title/Abstract]))) OR ((melanoma[Title/Abstract]) AND (common naevus[Title/Abstract]))) OR ((melanoma[Title/Abstract]) AND (common nevus[Title/Abstract]))) OR ((melanoma[Title/Abstract]) AND (acquired naevus[Title/Abstract]))) OR ((melanoma[Title/Abstract]) AND (acquired nevus[Title/Abstract]))) OR ((melanoma[Title/Abstract]) AND (dysplastic naevus[Title/Abstract]))) OR ((melanoma[Title/Abstract]) AND (dysplastic nevus[Title/Abstract]))) OR ((melanoma[Title/Abstract]) AND (dysplastic[Title/Abstract]))) OR (nevus associated melanoma[Title/Abstract])) OR (naevus-associated melanoma[Title/Abstract])) OR (nevus-associated melanoma[Title/Abstract])) OR (((melanoma[MeSH Terms]) AND (nevus[MeSH Terms] OR naevus[MeSH Terms]) AND ((remnant[All Fields] OR nevus-associated[All Fields] OR naevus-associated[All Fields])))

Supplementary Table S3. Among the 97 articles that were assessed full text, below are the reasons for the exclusion of 75 articles

AUTHOR	JOURNAL	YEAR	TITLE	DOI	REASON FOR EXCLUSION
	International Journal of Dermatology	2018	Erratum to: Recognition of early melanoma: a monocentric dermoscopy follow-up study comparing de novo melanoma with nevus-associated melanoma	10.1111/ijd.14089	Not original article. Erratum
Ahmed	Am J Dermatopatho l	1995	Interleukin (IL)-1 alpha- and -1 beta-, IL-6-, and tumor necrosis factor-alpha-like immunoreactivities in human common and dysplastic nevocellular nevi and malignant melanoma	10.1097/00000372-199506000-00002	Acquired melanocytic nevi types associated with NAM not reported
Ahmed	Br J Dermatol	1999	Expression of the neuronal isoform of nitric oxide synthase (nNOS) and its inhibitor, protein inhibitor of nNOS, in pigment cell lesions of the skin	10.1046/j.1365-2133.1999.02915.x	Acquired melanocytic nevi types associated with NAM not reported
Aloi	Giornale Italiano di Dermatologia e Venereologia 130(6), 357-363	1995	Melanoma arising in exophytic melanocytic nevus (Unna's nevus). A clinico-pathological study.	not available	Not retreivable

Alshedoukhy	Clin Transl Oncol	2020	A retrospective study of malignant melanoma from a tertiary care centre in Saudi Arabia from 2004 to 2016.	10.1007/s12094-019-02169-w	Acquired melanocytic nevi types associated with NAM not reported
Arndt	Jama	1984	Precursors to malignant melanoma: congenital and dysplastic nevi	10.1001/jama.1984.03340380064029	Not original article. Photo essay
Banky	Arch Dermatol	2005	Incidence of new and changed nevi and melanomas detected using baseline images and dermoscopy in patients at high risk for melanoma	10.1001/archderm.141.8.998	Acquired melanocytic nevi types associated with NAM not reported
Berentzen	Tidsskrift for den Norske Laegeforening,	2022	Melanom – utredning og primærbehandling.	10.4045/tidsskr.22.0043	Not english
Betti	J Eur Acad Dermatol Venereol,	2014	Observational study on the mitotic rate and other prognostic factors in cutaneous primary melanoma arising from naevi and from melanoma de novo	10.1111/jdv.12395	Acquired melanocytic nevi types associated with NAM not reported
Bevona	Arch Dermatol	2003	Cutaneous melanomas associated with nevi.	10.1001/archderm.139.12.1620	Common nevi reported together with congenital nevi in NAM
Bevona	Arch Dermatol	2003	Cutaneous melanomas associated with nevi.	10.1001/archderm.139.12.1620	duplicate

Bosch-Amate	Acta Derm Venereol	2021	Clinicopathological, Genetic and Survival Advantages of Naevus-associated Melanomas: A Cohort Study.	10.2340/00015555-3780	Acquired melanocytic nevi types associated with NAM not reported
Carli	J Am Acad Dermatol	1999	Cutaneous melanoma histologically associated with a nevus and melanoma de novo have a different profile of risk: results from a case-control study	10.1016/s0190-9622(99)70436-6	Acquired melanocytic nevi types associated with NAM not reported
Chiarugi	Journal of the European Academy of Dermatology and Venereology	2012	Familial and sporadic melanoma: Different clinical and histopathological features in the Italian population - A multicentre epidemiological study - By GIPMe (Italian Multidisciplinary Group on Melanoma)	10.1111/j.1468-3083.2011.04035.x	Acquired melanocytic nevi types associated with NAM not reported
Clark	Hum Pathol,	1998	Problems with lesions related to the development of malignant melanoma: common nevi, dysplastic nevi, malignant melanoma in situ, and radial growth phase malignant melanoma	10.1016/s0046-8177(98)90384-7	Not original article. Review
Colebatch	Pathology	2022	Elevated non-coding promoter mutations are associated with malignant transformation of	10.1016/j.pathol.2021.12.289	Acquired melanocytic nevi types associated with NAM not reported

			melanocytic naevi to melanoma		
Cook	Histopatholog y	1985	Melanocytic dysplasia and melanoma.	10.1111/j.1365-2559.1985.tb02845.x	Acquired melanocytic nevi types associated with NAM not reported
Crutcher	West J Med	1987	Dysplastic nevi-markers and precursors of malignant melanoma	no DOI	Not original article. Commentary
Cuevas-González	Dermatologia Revista Mexicana	2019	Frequency of skin cancer; experience of 10 years in a center of histopathological diagnosis in the city of Durango, Durango, México.	no DOI	Not english
Cymerman	J Natl Cancer Inst	2016	De Novo vs Nevus-Associated Melanomas: Differences in Associations With Prognostic Indicators and Survival.	10.1093/jnci/djw121	Acquired melanocytic nevi types associated with NAM not reported
De Giorgi	Melanoma Res	2020	Nevi and Breslow thickness in melanoma: sex differences?	10.1097/cmr.0000000000000579	NAM not reported
Dessinioti	British Journal of Dermatology,	2021	A multicentre study of naevus-associated melanoma vs. de novo melanoma, tumour thickness and body site differences	10.1111/bjd.19819	Acquired melanocytic nevi types associated with NAM not reported
Duray	Arch Dermatol	1987	Dysplastic nevus in histologic contiguity with acquired nonfamilial melanoma. Clinicopathologic experience in a 100-bed hospital	10.1001/archderm.1987.0166025008602 5	Less than 10 cases of NAM

Elder	Pathology	1985	The dysplastic nevus.	10.3109/00313028509063770	Not original article. Review
Elder	Pathology	2016	Melanoma progression.	10.1016/j.pathol.2015.12.002	Not original article. Review
Friedman	Arch Dermatol	1983	Favorable prognosis for malignant melanomas associated with acquired melanocytic nevi.	10.1001/archderm.1983.01650300009007	Acquired melanocytic nevi types associated with NAM not reported
Friedman	Dermatol Clin	1988	The relationship between melanocytic nevi and malignant melanoma	10.1016/S0733-8635(18)30671-5	Numbers of acqouired nevus types not clear. Unpublished data mentioned in a review paper.
Gassenmaier	Cancers (Basel)	2021	Diffuse PRAME Expression Is Highly Specific for Thin Melanomas in the Distinction from Severely Dysplastic Nevi but Does Not Distinguish Metastasizing from Non-Metastasizing Thin Melanomas.	10.3390/cancers13153864	Acquired melanocytic nevi types associated with NAM not reported
Ghiasvand	British Journal of Dermatology	2021	Divergent pathways for melanoma: what is the role of genetics?	10.1111/bjd.19887	Commentary
Giavina-Bianchi	An Bras Dermatol	2020	Worse survival of invasive melanoma patients in men and “de novo” lesions	10.1016/j.abd.2019.07.003	Acquired melanocytic nevi types associated with NAM not reported

Gibbs	J Natl Cancer Inst	2016	Association of interferon regulatory factor-4 polymorphism rs12203592 with divergent melanoma pathways.	10.1093/jnci/djw004	Acquired melanocytic nevi types associated with NAM not reported
Goodson	Dermatol Surg	2011	A decade of melanomas: identification of factors associated with delayed detection in an academic group practice.	10.1111/j.1524-4725.2011.02097.x	Acquired melanocytic nevi types associated with NAM not reported
Göppner	Acta Derm Venereol	2014	High incidence of naevi-associated BRAF wild-type melanoma and dysplastic naevi under treatment with the class I BRAF inhibitor vemurafenib	10.2340/00015555-1813	Less than 10 cases of NAM
Gruber	J Am Acad Dermatol	1989	Nevomelanocytic proliferations in association with cutaneous malignant melanoma: a multivariate analysis.		Common nevi reported together with congenital nevi in NAM
Hacker	J Invest Dermatol	2010	The association between MC1R genotype and BRAF mutation status in cutaneous melanoma: findings from an Australian population.	10.1038/jid.2009.182	Acquired melanocytic nevi types associated with NAM not reported
Hacker	J Invest Dermatol	2016	Histologic and Phenotypic Factors and MC1R Status Associated with BRAF(V600E),	10.1016/j.jid.2015.12.035	Acquired melanocytic nevi types associated with NAM not reported

			BRAF(V600K), and NRAS Mutations in a Community-Based Sample of 414 Cutaneous Melanomas.		
Haenssle	JAMA Dermatol,	2016	Association of Patient Risk Factors and Frequency of Nevus-Associated Cutaneous Melanomas	10.1001/jamadermatol.2015.3775	Acquired melanocytic nevi types associated with NAM not reported
Han	Biomed Res Int,	2020	dentification, Validation, and Functional Annotations of Genome-Wide Profile Variation between Melanocytic Nevus and Malignant Melanoma	10.1155/2020/1840415	Acquired melanocytic nevi types associated with NAM not reported
Hastrup	Am J Dermatopatho l	1991	The presence of dysplastic nevus remnants in malignant melanomas. A population-based study of 551 malignant melanomas	10.1097/00000372-199108000-00009	Acquired melanocytic nevi types associated with NAM not reported. Only dysplastic.
Helm	JAAD Case Rep	2021	Melanoma arising in a persistent nevus: Melanoma where 'pseudomelanoma' is expected	10.1016/j.jdcr.2021.03.045	Less than 10 cases of NAM. Case report
Kakavand	Pathology	2014	Concordant BRAFV600E mutation status in primary melanomas and associated naevi: Implications for mutation testing of primary melanomas.		Acquired melanocytic nevi types associated with NAM not reported

Klein	Aktuelle Dermatologie	2021	Two types of nevus-associated melanomas	10.1055/a-1389-0721	not english
Kolm	Giornale Italiano di Dermatologia e Venereologia	2010	Dermoscopy patterns of nevi associated with melanoma	145(1), 99-110.	Not original article. Review
Kraft	British Journal of Dermatology	2015	Melanoma-associated naevi: Precursors or coincidence?	10.1111/bjd.14059	Not original article. Editorial
Lai	Dermatol Pract Concept	2022	Prevalence and clinical-pathological features of nevus-associated versus de novo melanoma: a retrospective cross-sectional study of 2806 cases.	10.5826/dpc.1202a94	Acquired melanocytic nevi types associated with NAM not reported
Lin	J Am Acad Dermatol,	2015	Outcome of patients with de novo versus nevus-associated melanoma	10.1016/j.jaad.2014.09.028	Acquired melanocytic nevi types associated with NAM not reported
Martín-Gorgojo	Actas Dermosifiliogr	2018	Melanoma Arising in a Melanocytic Nevus.	10.1016/j.ad.2017.06.009	Not english
McDonald	Skin Health Dis	2021	Incidence of cutaneous melanoma in patients with histologically confirmed dysplastic naevus: A follow-up study in a large UK Healthcare Trust	10.1002/ski2.44	Acquired melanocytic nevi types associated with NAM not reported

Millán-esteban	Cancers (Basel)	2021	Mutational characterization of cutaneous melanoma supports divergent pathways model for melanoma development	10.3390/cancers13205219	Acquired melanocytic nevi types associated with NAM not reported
Murphy	J Biomed Opt,	2005	Toward the discrimination of early melanoma from common and dysplastic nevus using fiber optic diffuse reflectance spectroscopy	10.1117/1.2135799	Acquired melanocytic nevi types associated with NAM not reported
Pampena	Int J Dermatol	2020	Digital dermoscopic changes during follow-up of de-novo and nevus-associated melanoma: a cohort study	10.1111/ijd.14918	Acquired melanocytic nevi types associated with NAM not reported
Pampena	J Invest Dermatol,	2018	Nevus-Associated Melanoma: Patient Phenotype and Potential Biological Implications.	10.1016/j.jid.2018.01.025	Not original article. Commentary
Pan	Med J Aust	2017	Nodular melanoma is less likely than superficial spreading melanoma to be histologically associated with a naevus	10.5694/mja17.00232	Acquired melanocytic nevi types associated with NAM not reported
Pandeya	J Invest Dermatol	2018	Factors Related to Nevus-Associated Cutaneous Melanoma: A Case-Case Study.	10.1016/j.jid.2017.12.036	Acquired melanocytic nevi types associated with NAM not reported
Prieto Herman Reinehr	Dermatology	2022	Dermoscopic Nevus Patterns Associated with Melanoma Patients.	10.1159/000520164	Acquired melanocytic nevi types associated with NAM not reported

Purdue	Cancer Epidemiol Biomarkers Prev	2005	Etiologic and other factors predicting nevus-associated cutaneous malignabt melanoma	https://doi.org/10.1158/1055-9965.epi-05-0097	Acquired melanocytic nevi types associated with NAM not reported
Rashid	JID Innov	2021	Unsupervised Phenotype-Based Clustering of Clinicopathologic Features in Cutaneous Melanoma	10.1016/j.xjidi.2021.100047	Acquired melanocytic nevi types associated with NAM not reported
Reddy	JAMA Dermatol	2013	Atypical (dysplastic) nevi: outcomes of surgical excision and association with melanoma	10.1001/jamadermatol.2013.4440	Acquired melanocytic nevi types associated with NAM not reported
Reiter	J Eur Acad Dermatol Venereol	2021	The differences in clinical and dermoscopic features between in situ and invasive nevus-associated melanomas and de novo melanomas.	10.1111/jdv.17133	Acquired melanocytic nevi types associated with NAM not reported
Rezze	Acta Derm Venereol	2012	Primary cutaneous melanoma arising in agminated melanocytic nevi: CDKN2A and CDK4 mutation screening	10.2340/00015555-1165	Less than 10 cases of NAM. Case report
Rhodes	Ann Intern Med	1985	Acquired dysplastic melanocytic nevi and cutaneous melanoma: precursors and prevention	10.7326/0003-4819-102-4-546	Not original article. Review
Rigel	Cancer	1989	Dysplastic nevi. Markers for increased risk for melanoma	10.1002/1097-0142	LESS THAN 10 CASES OF NAM
Scalvenzi	Australas J Dermatol	2020	Cutaneous melanoma associated with naevi	10.1111/ajd.13171	Acquired melanocytic nevi types associated with NAM not reported

			prevalence: A 15-year cross-sectional retrospective study.		
Shitara	Acta Derm Venereol	2015	Dermoscopy of naevus-associated melanomas.	10.2340/00015555-2009	Acquired melanocytic nevi types associated with NAM not reported
Shitara	Am J Clin Pathol	2014	Nevus-associated melanomas: clinicopathologic features.	10.1309/ajcp4l5cjgktjvdd	Acquired melanocytic nevi types associated with NAM not reported
Shitara	Br J Dermatol,	2016	Discrepant mutational status between naevi and melanomas in naevus-associated melanomas: about mutation-specific immunohistochemistry: reply from the authors	10.1111/bjd.14613	Not original article. Reply
Stante	Clin Exp Dermatol	2003	Dermoscopic features of naevus-associated melanoma.	10.1046/j.1365-2230.2003.01332.x	Acquired melanocytic nevi types associated with NAM not reported
Stolz	Cancer	1989	Association of early malignant melanoma with nevocytic nevi	10.1002/1097-0142(19890201)63:3%3C550::aid-cncr2820630325%3E3.0.co;2-a	Acquired dysplastic melanocytic nevi types associated with NAM not reported
Tan	Clin Exp Dermatol,	2018	Association of clinicopathological features of melanoma with total naevus count and a history of dysplastic naevi: a cross-sectional retrospective study within an academic centre	10.1111/ced.13393	Acquired melanocytic nevi types associated with NAM not reported

Tas	Pathol Oncol Res	2020	De Novo and Nevus-Associated Melanomas: Different Histopathologic Characteristics but Similar Survival Rates.	10.1007/s12253-020-00858-4	Acquired melanocytic nevi types associated with NAM not reported
Tschandl	PLOS One	2013	NRAS and BRAF mutations in melanoma-associated nevi and uninvolved nevi		Common nevi reported together with congenital nevi in NAM
Vu	British Journal of Dermatology	2018	Impact of naevus association on survival for nodular and superficial spreading melanomas.	10.1111/bjd.16556	Acquired melanocytic nevi types associated with NAM not reported
Weatherhead	British Journal of Dermatology	2007	Melanomas arising from naevi and de novo melanomas - Does origin matter?	10.1111/j.1365-2133.2006.07570.x	Acquired melanocytic nevi types associated with NAM not reported
Wiggins	Br J Dermatol	2021	Melanoma origins: data from early-stage tumours supports de novo and naevus-associated melanomas as distinct subtypes	10.1111/bjd.20396	commentary
Wood	Cancer Epidemiology Biomarkers and Prevention	2020	Association of Known Melanoma Risk Factors with Primary Melanoma of the Scalp and Neck.	10.1158/1055-9965.EPI-20-0595	10.1158/1055-9965.EPI-20-0595

Supplementary Table S4. Assessment of risk of bias of included studies. Details of studies are presented in Table 1.

**Study**	**Study design**	**Inconsistency** (study in not relevant subgroups, e.g. invasive or in situ, histological subtypes)	**Imprecision** (High risk for <30 NAMs with acquired nevi)	**Overall risk of bias**
Alendar, 2018[1]	Consecutive patients	Moderate	High	High
Alvarez Martinez, 2018[2]	Preselected invasive melanomas with 2 follow-up images	High	High	High
Black, 1988[3]	Invasive SSMs	Low	Low	Low
Bogdan, 2003[4]	Preselected NAM	Moderate	Moderate	Moderate
Clark, 1984[5]	Consecutive patients	Low	Low	Low
Duman, 2015[6]		High	High	High
Harley, 1996[7]	Consecutive patients	High	High	High
Kaddu, 2002[8]	Consecutive patients	Low	Low	Low
Kamino, 2009[9]	Preselected NAM of SSM or NM subtype	Low	Moderate	Moderate
Longo, 2011[10]	Consecutive patients with confocal images	High	High	High
Manganoni, 2010[11]	Consecutive patients with NM	High	High	High
Manrique-Silva, 2019[12]	Patients with invasive SSM or NM, and NAM with common or dysplastic nevi	Low	Low	Low
Marks, 1990[13]	Patients with SSM or NM	Low	Low	Low
Martin-Gorgojo, 2018[14]	Invasive melanoma, NAM with common or dysplastic nevi	Low	Low	Low
Massi, 1999[15]	Consecutive patients with invasive melanoma	Low	High	Moderate
Pampin-Franco, 2020[16]	Selected NAM with dermoscopy and confocal images	Moderate	High	High
Sagebiel, 1993[17]	Consecutive patients with SSM or NM	Low	Low	Low

Sheen, 2017[18]	Consecutive patients in Asia	High	High	High
Shitara, 2015[19]	Preselected NAM	Low	High	Moderate
Skender-Kalnenas, 1995[20]	Thin melanomas	Low	Low	Low
Smolle, 1999[21]	Consecutive melanoma biopsies	Moderate	Low	Moderate
Suhonen, 2021[22]	In 337 melanomas, information for nevus presence in 146 (43.3%)	Moderate	Low	Moderate

Supplementary Figure S1. Forest plot of the proportion of dysplastic nevus in NAM stratified by the risk of bias of individual studies. [1-22]

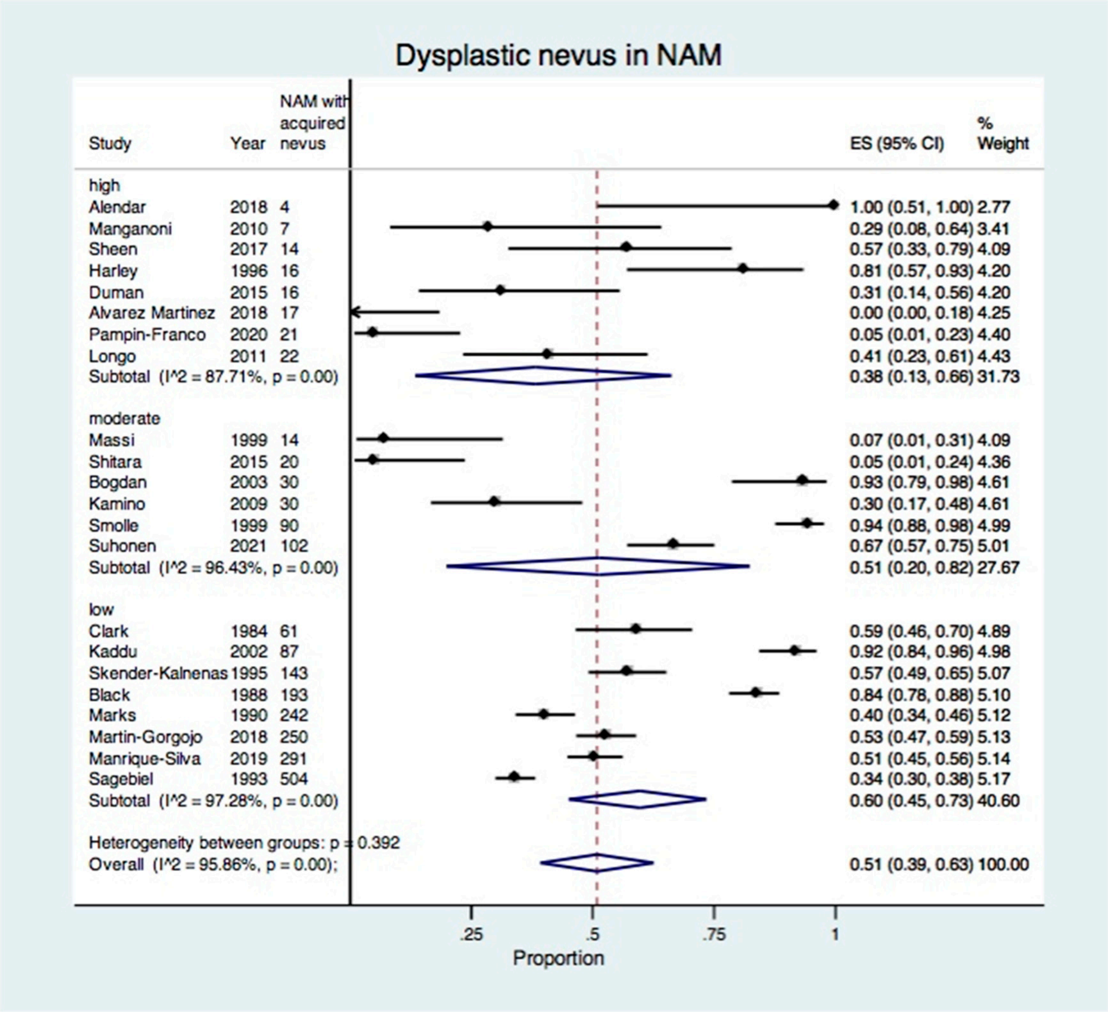

Supplementary Figure S2. Forest plot of the proportion of dysplastic nevus in studies with invasive only acquired nevus-NAM. [2,3,6,9,11,12,14,15]

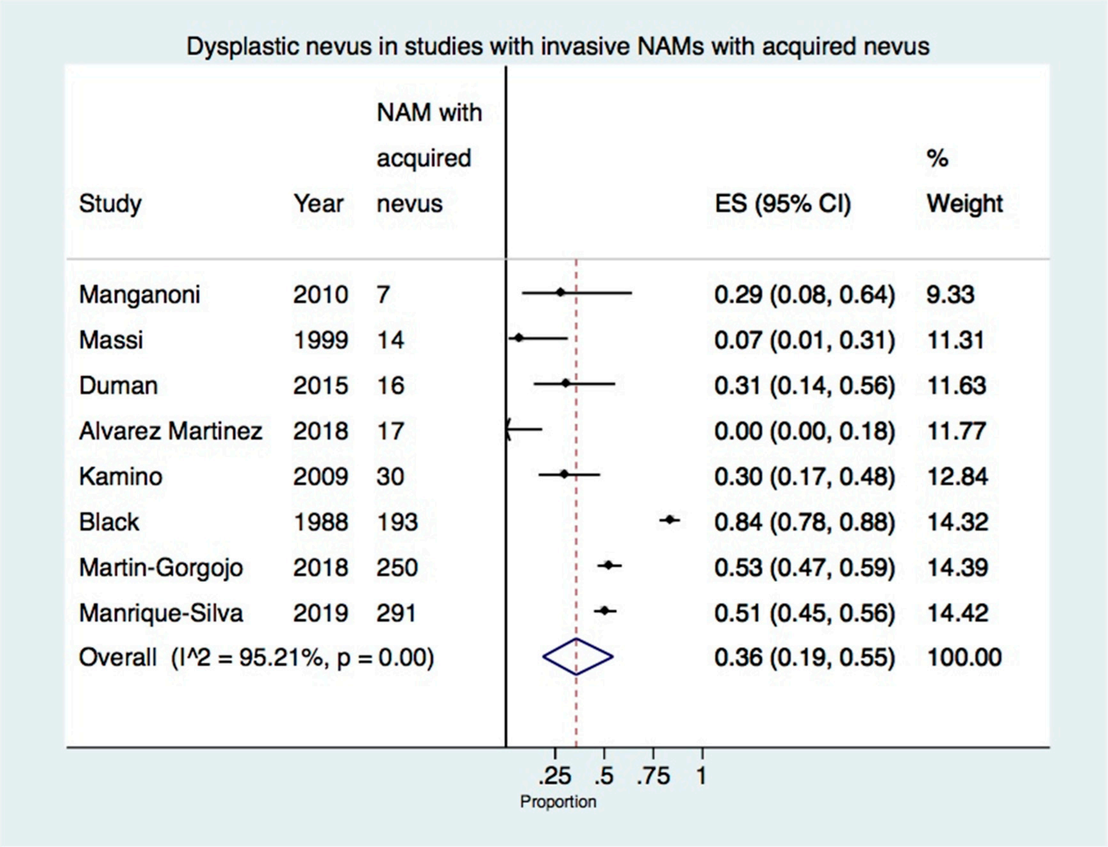

Supplementary Figure S3. Forest plot of the proportion of dysplastic nevus in acquired nevus-NAM, stratified by region. [1-18,20-22]

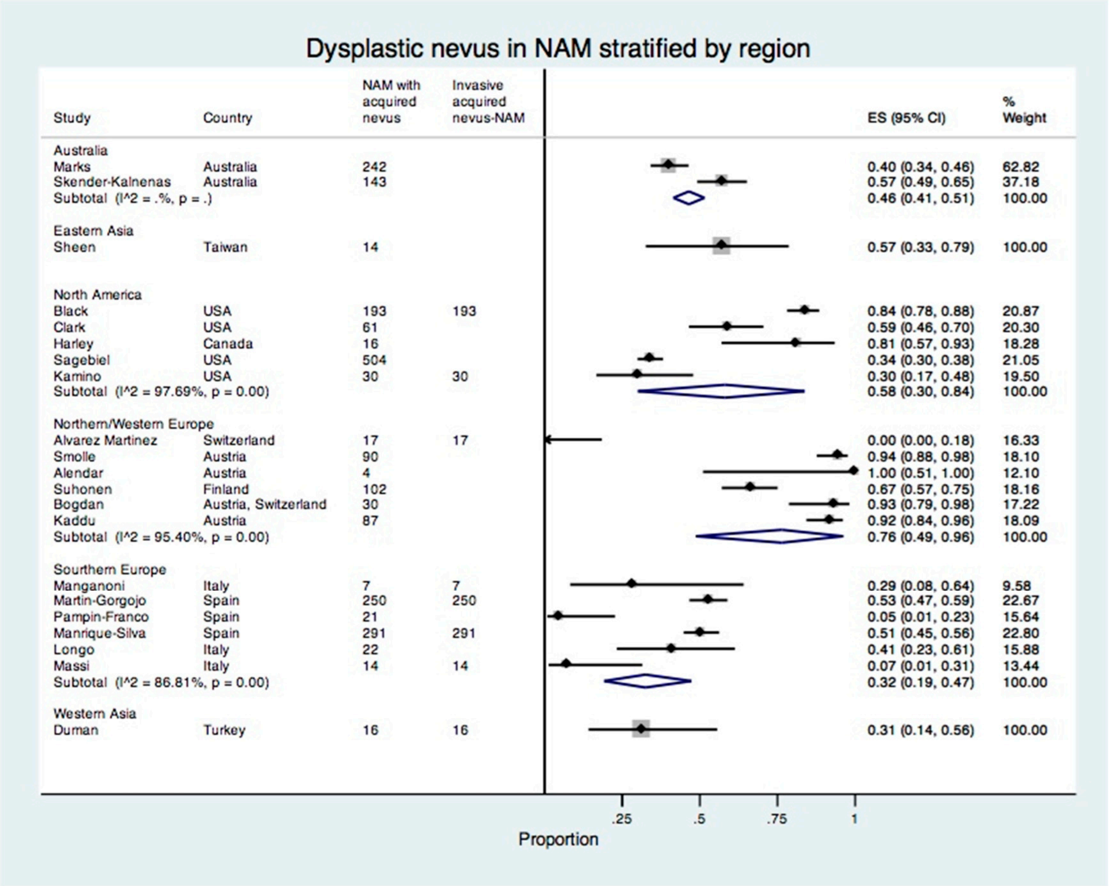

References for Supplementary material

1. Alendar, T.; Kittler, H. Morphologic characteristics of nevi associated with melanoma: a clinical, dermatoscopic and histopathologic analysis. *Dermatol Pract Concept* **2018**, *8*, 104-108, doi:10.5826/dpc.0802a07.
2. Alvarez Martinez, D.; Boehncke, W.H.; Kaya, G.; Merat, R. Recognition of early melanoma: a monocentric dermoscopy follow-up study comparing de novo melanoma with nevus-associated melanoma. *International journal of dermatology* **2018**, *57*, 692-702, doi:10.1111/ijd.13977.
3. Black, W.C. Residual dysplastic and other nevi in superficial spreading melanoma. Clinical correlations and association with sun damage. *Cancer* **1988**, *62*, 163-173, doi:10.1002/1097-0142(19880701)62:1<163::aid-cncr2820620126>3.0.co;2-n.

4. Bogdan, I.; Smolle, J.; Kerl, H.; Burg, G.; Boni, R. Melanoma ex naevo: a study of the associated naevus. *Melanoma Res* **2003**, *13*, 213-217, doi:10.1097/01.cmr.0000056226.78713.99.
5. Clark, W.H., Jr.; Elder, D.E.; Guerry, D.t.; Epstein, M.N.; Greene, M.H.; Van Horn, M. A study of tumor progression: the precursor lesions of superficial spreading and nodular melanoma. *Hum Pathol* **1984**, *15*, 1147-1165, doi:10.1016/s0046-8177(84)80310-x.
6. Duman, N.; Erkin, G.; Gokoz, O.; Karahan, S.; Kayikcioglu, A.U.; Celik, I. Nevus-Associated versus de novo Melanoma: Do They Have Different Characteristics and Prognoses? *Dermatopathology (Basel)* **2015**, *2*, 46-51, doi:10.1159/000375490.
7. Harley, S.; Walsh, N. A new look at nevus-associated melanomas. *Am J Dermatopathol* **1996**, *18*, 137-141, doi:10.1097/00000372-199604000-00005.
8. Kaddu, S.; Smolle, J.; Zenahlik, P.; Hofmann-Wellenhof, R.; Kerl, H. Melanoma with benign melanocytic naevus components: reappraisal of clinicopathological features and prognosis. *Melanoma Res* **2002**, *12*, 271-278, doi:10.1097/00008390-200206000-00011.
9. Kamino, H.; Tam, S.; Tapia, B.; Toussaint, S. The use of elastin immunostain improves the evaluation of melanomas associated with nevi. *Journal of cutaneous pathology* **2009**, *36*, 845-852, doi:10.1111/j.1600-0560.2008.01170.x.
10. Longo, C.; Rito, C.; Beretti, F.; Cesinaro, A.M.; Pineiro-Maceira, J.; Seidenari, S.; Pellacani, G. De novo melanoma and melanoma arising from pre-existing nevus: in vivo morphologic differences as evaluated by confocal microscopy. *J Am Acad Dermatol* **2011**, *65*, 604-614, doi:10.1016/j.jaad.2010.10.035.
11. Manganoni, A.M.; Farisoglio, C.; Gavazzoni, F.; Facchetti, F.; Zanotti, F.; Calzavara-Pinton, P. Nodular melanomas associated with nevi. *J Am Acad Dermatol* **2010**, *63*, e97, doi:10.1016/j.jaad.2009.12.039.
12. Manrique-Silva, E.; Reyes-Garcia, D.; Folgado, B.; Martin-Gorgojo, A.; Traves, V.; Requena, C.; Nagore, E. The proportion of nevus-associated invasive melanoma differs with Breslow thickness: A cross-sectional study of 1087 cutaneous melanomas. *J Am Acad Dermatol* **2019**, *81*, 852-854, doi:10.1016/j.jaad.2019.04.043.
13. Marks, R.; Dorevitch, A.P.; Mason, G. Do all melanomas come from "moles"? A study of the histological association between melanocytic naevi and melanoma. *The Australasian journal of dermatology* **1990**, *31*, 77-80, doi:10.1111/j.1440-0960.1990.tb00656.x.
14. Martin-Gorgojo, A.; Requena, C.; Garcia-Casado, Z.; Traves, V.; Kumar, R.; Nagore, E. Dysplastic vs. Common Naevus-associated vs. De novo Melanomas: An Observational Retrospective Study of 1,021 Patients. *Acta Derm Venereol* **2018**, *98*, 556-562, doi:10.2340/00015555-2908.
15. Massi, D.; Carli, P.; Franchi, A.; Santucci, M. Naevus-associated melanomas: cause or chance? *Melanoma Res* **1999**, *9*, 85-91, doi:10.1097/00008390-199902000-00011.
16. Pampin-Franco, A.; Gamo-Villegas, R.; Floristan-Muruzabal, U.; Pinedo-Moraleda, F.J.; Perez-Fernandez, E.; Garcia-Zamora, E.; Lopez-Estebaranz, J.L. Nevus-associated melanoma: An observational retrospective study of 22

patients evaluated with dermoscopy and reflectance confocal microscopy. *Skin Res Technol* **2020**, *26*, 99-104, doi:10.1111/srt.12770.

17. Sagebiel, R.W. Melanocytic nevi in histologic association with primary cutaneous melanoma of superficial spreading and nodular types: effect of tumor thickness. *J Invest Dermatol* **1993**, *100*, 322S-325S, doi:10.1111/1523-1747.ep12470218.
18. Sheen, Y.S.; Liao, Y.H.; Lin, M.H.; Chen, J.S.; Liau, J.Y.; Liang, C.W.; Chang, Y.L.; Chu, C.Y. Clinicopathological features and prognosis of patients with de novo versus nevus-associated melanoma in Taiwan. *PloS one* **2017**, *12*, e0177126, doi:10.1371/journal.pone.0177126.
19. Shitara, D.; Tell-Marti, G.; Badenas, C.; Enokihara, M.M.; Alos, L.; Larque, A.B.; Michalany, N.; Puig-Butille, J.A.; Carrera, C.; Malvehy, J.; et al. Mutational status of naevus-associated melanomas. *Br J Dermatol* **2015**, *173*, 671-680, doi:10.1111/bjd.13829.
20. Skender-Kalnenas, T.M.; English, D.R.; Heenan, P.J. Benign melanocytic lesions: risk markers or precursors of cutaneous melanoma? *J Am Acad Dermatol* **1995**, *33*, 1000-1007, doi:10.1016/0190-9622(95)90294-5.
21. Smolle, J.; Kaddu, S.; Kerl, H. Non-random spatial association of melanoma and naevi--a morphometric analysis. *Melanoma Res* **1999**, *9*, 407-412, doi:10.1097/00008390-199908000-00011.
22. Suhonen, V.; Rummukainen, J.; Siiskonen, H.; Mannermaa, A.; Harvima, I.T. High regional mortality due to malignant melanoma in Eastern Finland may be explained by the increase in aggressive melanoma types. *BMC Cancer* **2021**, *21*, 1155, doi:10.1186/s12885-021-08879-1.
